# 18F-FDG PET/CT metrics-based stratification of large B-cell lymphoma receiving CAR-T cell therapy: immunosuppressive tumor microenvironment as a negative prognostic indicator in patients with high tumor burden

**DOI:** 10.1186/s40364-024-00650-5

**Published:** 2024-09-14

**Authors:** Ling-Shuang Sheng, Rong Shen, Zi-Xun Yan, Chao Wang, Xin Zheng, Yi-Lun Zhang, Hao-Xu Yang, Wen Wu, Peng-Peng Xu, Shu Cheng, Emmanuel Bachy, Pierre Sesques, Nicolas Jacquet-Francillon, Xu-Feng Jiang, Wei-Li Zhao, Li Wang

**Affiliations:** 1 Shanghai Institute of Hematology, State Key Laboratory of Medical Genomics, National Research Center for Translational Medicine at Shanghai, Ruijin Hospital, Shanghai Jiao Tong University School of Medicine, Shanghai, China; 2 Department of Nuclear Medicine, Ruijin Hospital, Shanghai Jiao Tong University School of Medicine, Shanghai, China; 3 Department of Haematology, Hospices Civils de Lyon, Lyon, France; 4 Department of Nuclear Medicine, Hospices Civils de Lyon, Lyon, France

**Keywords:** Large B-cell lymphoma, CAR-T cell therapy, 18F-FDG PET/CT, Microenvironment

## Abstract

Chimeric antigen receptor T (CAR-T) cell therapy has greatly improved the prognosis of relapsed and refractory patients with large B-cell lymphoma (LBCL). Early identification and intervention of patients who may respond poorly to CAR-T cell therapy will help to improve the efficacy. Ninety patients from a Chinese cohort who received CAR-T cell therapy and underwent 18F-fluorodeoxyglucose positron emission tomography/computed tomography (18F-FDG PET/CT) scans at the screening stage (median time to infusion 53.5 days, range 27–176 days), 1 month and 3 months after CAR-T cell infusion were analyzed, with RNA-sequencing conducted on 47 patients at the screening stage. Patients with maximum diameter of the largest lesion (Dmax) < 6 cm (*N* = 60) at screening stage showed significantly higher 3-month complete response rate (85.0% vs. 33.3%, *P* < 0.001), progression-free survival (HR 0.17; 95% CI 0.08–0.35, *P* < 0.001) and overall survival (HR 0.18; 95% CI 0.08–0.40, *P* < 0.001) than those with Dmax ≥ 6 cm (*N* = 30). Besides, at the screening stage, Dmax combined with extranodal involvement was more efficient in distinguishing patient outcomes. The best cut-off values for total metabolic tumor volume (tMTV) and total lesion glycolysis (tTLG) at the screening stage were 50cm^3^ and 500 g, respectively. A prediction model combining maximum standardized uptake value (SUVmax) at 1 month after CAR-T cell therapy (M1) and tTLG clearance rate was established to predict early progression for partial response/stable disease patients evaluated at M1 after CAR-T cell therapy and validated in Lyon cohort. Relevant association of the distance separating the two farthest lesions, standardized by body surface area to the severity of neurotoxicity (AUC = 0.74; *P* = 0.034; 95% CI, 0.578–0.899) after CAR-T cell therapy was found in patients received axicabtagene ciloleucel. In patients with Dmax ≥ 6 cm, RNA-sequencing analysis conducted at the screening stage showed enrichment of immunosuppressive-related biological processes, as well as increased M2 macrophages, cancer-associated fibroblasts, myeloid-derived suppressor cells, and intermediate exhausted T cells. Collectively, immunosuppressive tumor microenvironment may serve as a negative prognostic indicator in patients with high tumor burden who respond poorly to CAR-T cell therapy.

**Supplementary Information:**

The online version contains supplementary material available at 10.1186/s40364-024-00650-5.

## To the editor

Chimeric Antigen Receptor T (CAR-T) cell therapy has greatly improved the prognosis of relapsed and refractory patients with large B-cell lymphoma (LBCL), with objective remission rate (ORR) as 83%, complete response (CR) as 58%, and 5-year overall survival (OS) as 42% [1]. Despite its efficacy, a subset of patients still experiences disease progression. Recently, it has been demonstrated that pre-apheresis and pre-infusion total metabolic tumor volume (tMTV) could predict survival and that higher pre-apheresis or pre-infusion tMTV values were associated with shorter progression-free survival (PFS) and OS. Furthermore, at pre-infusion, tMTV was associated with grade ≥ 2 cytokine release syndrome (CRS), and maximum standardized uptake value (SUVmax) was associated with failure to achieve CR. A predictive model using pre-infusion tMTV combined with lactate dehydrogenase (LDH) was established to predict patient outcomes after CAR-T cell therapy [2].

**Fig. 1:**
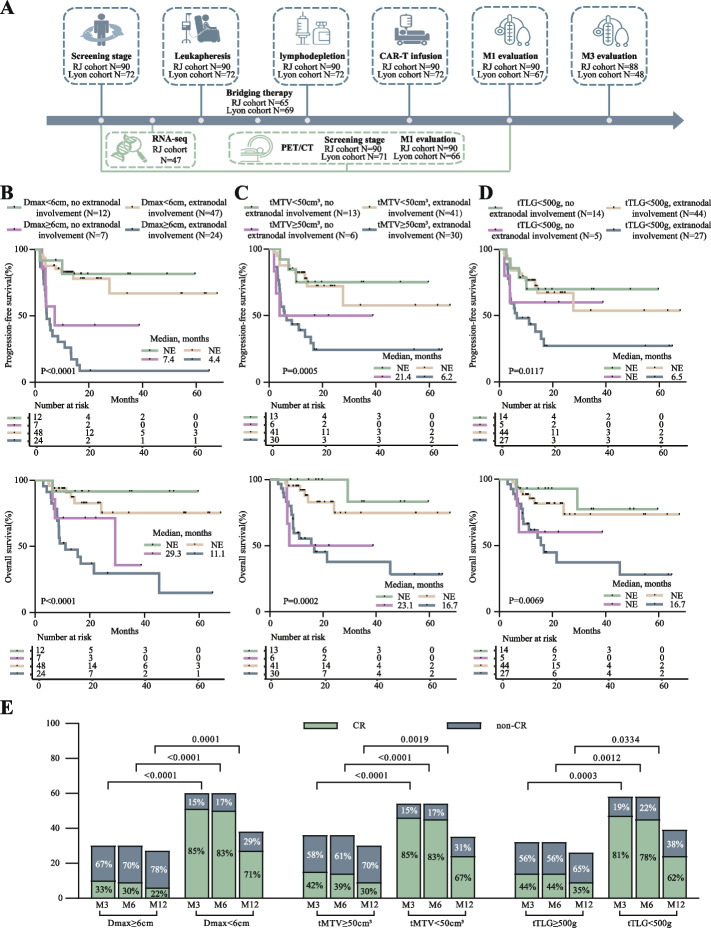
The value of screening-phase 18F-FDG PET/CT metrics in predicting response, prognosis, and death. **A** Examination time points during CAR-T cell therapy process. **B**-**D** PFS and OS of patients stratified with whether extranodal involvement and screening-phase Dmax (**B**), tMTV (**C**), and tTLG (**D**). **E** 3-, 6- and 12-month responses after CAR-T cell therapy stratified with screening-phase 18F-FDG PET/CT metrics. 18F-FDG PET/CT, 18F-fluorodeoxyglucose positron emission tomography/computed tomography; CAR-T, chimeric antigen receptor T; PFS, progression-free survival; OS, overall survival; Dmax, the maximum diameters of the largest lesion; tMTV, total metabolic tumor volume; tTLG, total lesion glycolysis

Here we reviewed 90 Chinese LBCL patients (53 males, 37 females; median age 56.5 years) received CAR-T cell therapy at our institution from January 1, 2018 to March 31, 2023 (baseline characteristics showed in Table 1). Key radiomic metrics included SUVmax, tMTV, total lesion glycolysis (tTLG), maximum diameter of the largest lesion (Dmax), and distance separating the two farthest lesions, standardized by body surface area (hereafter referred to as distance of the farthest lesions), at screening or 1-month after CAR-T cell infusion. By analyzing these radiomics metrics in conjunction with the M3 response and survival of patients, patients at screening with Dmax < 6cm (*N* = 60) had higher 3-month CR rates (85.0% vs. 33.3%, *P* < 0.001), PFS (HR 0.17; 95% CI 0.08–0.35, *P* < 0.001), and OS (HR 0.18; 95% CI 0.08–0.40, *P* < 0.001). Similarly, patients with tMTV < 50cm^3^ and tTLG < 500g had higher 3-month CR rates, PFS and OS (Fig. 1). Based on the areas under curve (AUC) of the receiver operating characteristic (ROC) curves for each metric and multivariate analysis, Dmax had the largest AUC and the most significant P-value, therefore, it was the optimal screening metric for predicting prognosis (Supplementary Fig. 1A-C and Table 2). Next, we validated previously reported predictor model [2] using our cohort and found that Dmax combined with extranodal involvement was more efficient in distinguishing patient outcome than the combination of tMTV or tTLG with extranodal involvement or LDH (Fig. 1B-D and Table 2).

**Table 1:** Baseline characteristics of RJ cohort and Lyon cohort

		**RJ cohort (*n* = 90)**	**Lyon cohort (*N* = 72)**
**Age**	> 60	31 (34.4%)	33 (45.8%)
	≤ 60	59 (65.6%)	39 (54.2%)
**Gender**	Male	53 (58.9%)	44 (61.1%)
	Female	37 (41.1%)	28 (38.9%)
**ECOG score**	0–1	63 (70.0%)	53 (73.6%)
	≥ 2	27 (30.0%)	19 (26.4%)
**Ann Arbor stage**	I-II	21 (23.3%)	18 (25.0%)
	III-IV	69 (76.7%)	54 (75.0%)
**LDH level**	Normal	19 (21.1%)	16 (22.2%)
	Elevated	71 (78.9%)	56 (77.8%)
**Extranodal sites**	0–1	36 (40.0%)	NA
	≥ 2	54 (60.0%)	NA
**IPI score**	0–2	33 (36.7%)	NA
	3–5	57 (63.3%)	NA
**Disease type**	DLBCL	72 (80.0%)	45 (62.5%)
	PBMCL	4 (4.4%)	5 (6.9%)
	PCNSL	1 (1.1%)	0
	Transformed low-grade lymphoma	13 (14.4%)	22 (30.6%)
**Cell of origin**	GCB	35 (38.9%)	36/63 (57.1%)
	Non-GCB	55 (61.1%)	27/63 (42.9%)
**Prior lines of therapy**	1–2	56 (62.2%)	17 (23.6%)
	≥ 3	34 (37.8%)	55 (76.4%)
**Primary refractory**	No	26 (28.9%)	25 (34.7%)
	Yes	64 (71.1%)	47 (65.3%)
**Response to last line**	PR	19 (21.1%)	NA
	SD/PD	71 (78.9%)	NA
**Prior ASCT**	No	83 (92.2%)	52 (72.2%)
	Yes	7 (7.8%)	20 (27.8%)
**Bulky disease ≥ 6cm**	No	60 (66.7%)	37/59 (62.7%)
	Yes	30 (33.3%)	22/59 (37.3%)
**Double expressor**	No	52 (57.8%)	20/64 (31.2%)
	Yes	38 (42.2%)	44/64 (68.8%)
**Double/triple-hit**	No	79 (87.8%)	26/31 (83.9%)
	Yes	11 (12.2%)	5/31 (16.1%)
**TP53 mutation**	No	59 (65.6%)	NA
	Yes	31 (34.4%)	NA
**CAR-T products**	Axi-cel	61 (67.8%)	33 (45.8%)
	Relma-cel	29 (32.2%)	0
	Kymriah	0	39 (54.2%)

**Table 2:** Univariate and multivariate logistic regression of the predictive factors for progression-free survival

**Characteristics**	**Univariate analysis**		**Multivariate analysis**	
	**OR (95%CI)**	***P* value**	**OR (95%CI)**	***P* value**
Age > 60	1.053 (0.436–2.546)	0.908	0.535 (0.115–2.496)	0.426
Male	1.041 (0.443–2.445)	0.927	0.485 (0.116–2.031)	0.322
ECOG ≥ 2	1.509 (0.607–3.749)	0.376	0.918 (0.197–4.283)	0.913
Ann Arbor stage ≥ 3	1.538 (0.552–4.286)	0.410	0.931 (0.185–4.681)	0.931
LDH elevated	0.949 (0.340–2.650)	0.921	0.351 (0.076–1.627)	0.181
Extranodal involvement	1.679 (0.573–4.920)	0.345	4.376 (0.805–23.792)	0.087^*^
GCB subtype	0.511 (0.210–1.243)	0.511	0.376 (0.094–1.506)	0.167
Prior lines of therapy ≥ 3	1.220 (0.514–2.894)	0.652	1.543 (0.370–6.438)	0.551
Primary refractory	1.168 (0.459–2.968)	0.745	1.104 (0.265–4.596)	0.892
Response to last line: SD/PD	2.297 (0.747–7.063)	0.147	2.473 (0.531–11.527)	0.249
Prior ASCT	1.081 (0.227–5.141)	0.922	0.726 (0.070–7.558)	0.789
Double expressor	1.074 (0.459–2.510)	0.870	1.545 (0.410–5.830)	0.521
Product: Axi-cel	0.648 (0.265–1.585)	0.342	0.556 (0.130–2.384)	0.429
Dmax ≥ 6cm	20.000 (6.334–63.163)	0.000^**^	25.178 (4.958–127.861)	0.000^**^
tMTV ≥ 50cm^3^	6.308 (2.483–16.025)	0.000^**^	2.085 (0.247–17.630)	0.500
tTLG ≥ 500g	4.020 (1.615–10.007)	0.003^**^	1.265 (0.145–11.0.28)	0.832

*Abbreviations*: *ECOG* Eastern Cooperative Oncology Group, *LDH* lactate dehydrogenase, *IPI* international prognostic index, *DLBCL* diffuse large B-cell lymphoma, *PMBCL* primary mediastinal B-cell lymphoma, *PCNSL* primary central nervous system lymphoma, *GCB* germinal center B cell, *PR* partial response, *SD* stable disease, *PD* progressive disease, *ASCT* autologous stem-cell transplantation, *CAR-T* chimeric antigen receptor T, *Axi-cel* axicabtagene ciloleucel, *Relma-cel* relmacabtagene autoleucel, *PFS* progression-free survival, *OR* odds ratio, *CI* confidential interval

^*^P < 0.1

^**^P < 0.05

Identifying patients who will experience early progression is critical to implementing preemptive treatment strategies. Therefore, we developed a prediction model for early progression using these radiomic data for partial response (PR)/ stable disease (SD) patients 1 month after CAR-T cell therapy. Recently, growing evidence has demonstrated that high SUVmax in M1 is strongly associated with poor prognosis [3–5]. Of note, the tTLG index is derived in conjunction with lesion volume and spatial distribution to measure the metabolic activity of the lesion and treatment response. We found that the prediction model combining the SUVmax and the tTLG clearance rate (ΔtTLG / tTLG^pre^ = [tTLG at screening stage]-[tTLG at M1]) / tTLG at screening stage) of M1 was able to accurately predict the patients without progression after CAR-T cell therapy. M1 SUVmax < 8 and ΔtTLG / tTLG^pre^ ≥ 0.9 (*N* = 11, median PFS not reached) predicted a constant state of remission. However, SUVmax ≥ 8 indicted progression within 6 months, regardless of tTLG clearance rate (tTLG clearance rate ≥ 0.9, *N* = 6, median PFS 4.2 months, HR 95.0, 95%CI 13.6–665.6 months; tTLG clearance rate < 0.9, *N* = 7, median PFS 3.7 months, HR 65.2, 95%CI 11.3–376.0 months), while M1 SUVmax < 8 and tTLG clearance rate < 0.9 (*N* = 5, median PFS 4.4 months, HR 69.6, 95%CI 8.8–551.2 months) indicated progression in 12 months (Supplementary Fig. 2A, 2E). Furthermore, this model was validated by Lyon cohort (Supplementary Fig. 2B) [4], indicating that the model is robust and reliable, as the genetic background and ethnicity of patients do not affect the predictive power of the model.

As for the predictive value of adverse events during CAR-T cell therapy, a correlation between baseline MTV with CRS/neurotoxicity (NT) grades [6–8], and baseline SUVmax with CRS [3] has been revealed. In our cohort, for axicabtagene ciloleucel (axi-cel), distance of the farthest lesions was associated with NT (AUC = 0.74) (Supplementary Fig. 3). No NT occurred in patients with distance of the farthest lesions < 0.15m^−1^, while 34.8% of patients with distance of the farthest lesion ≥ 0.15m^−1^ experienced NT. However, for relmacabtagene autoleucel (relma-cel), no strong correlations were observed. Imaging metrics did not correlate significantly with CRS/NT duration or onset, or with CAR-T cell expansion metrics (Supplementary Fig. 4). Therefore, incidence of CRS and NT could vary from different CAR-T cells, probably due to differences in CAR-T cell co-stimulatory molecules and could also be due to the small sample size.

**Fig. 2:**
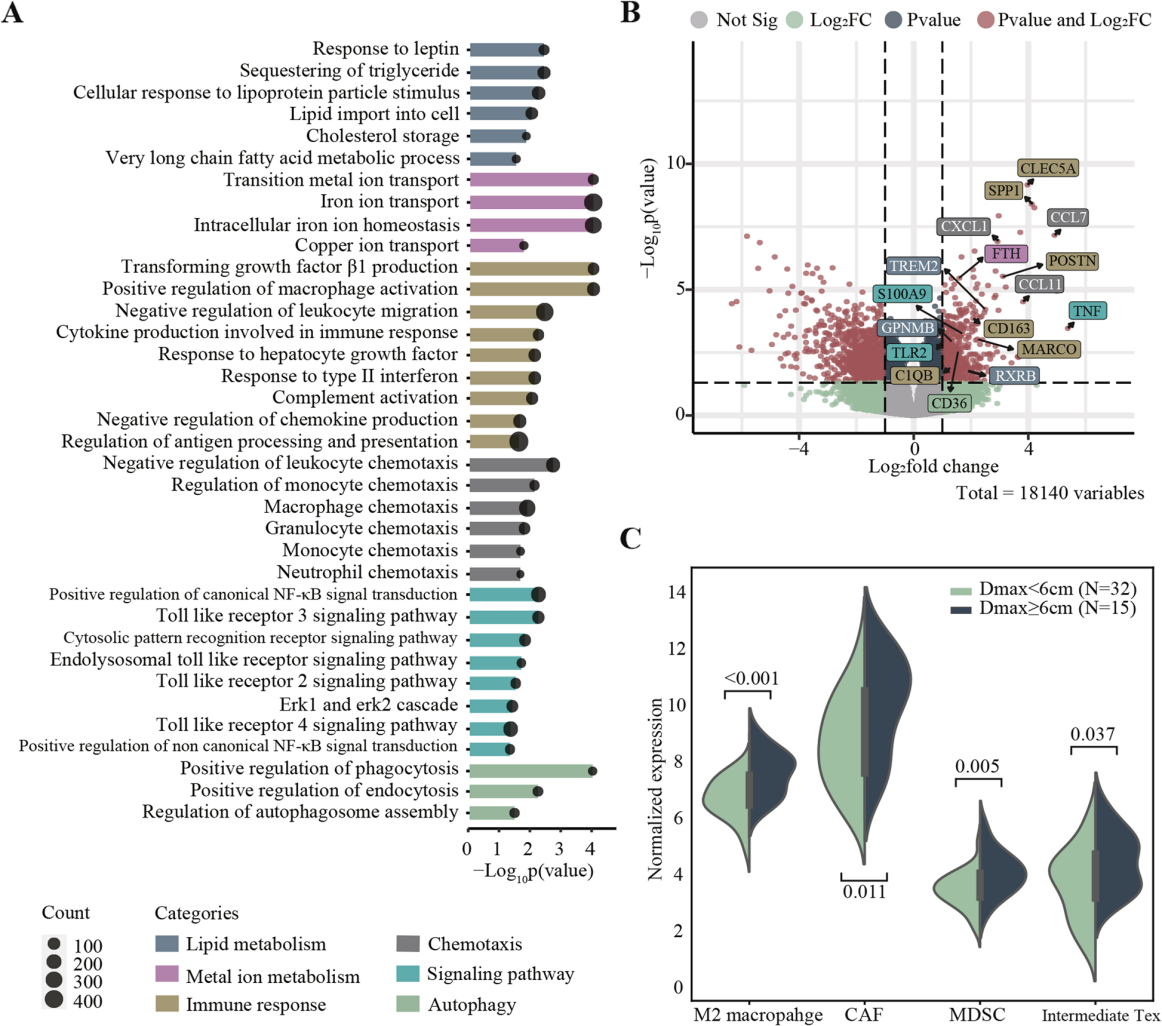
Biological process and tumor microenvironment characteristics in patients stratified with Dmax. **A** Enriched BP terms in patients with Dmax ≥ 6 cm, as compared to patients with Dmax < 6 cm in CAR-T screening phase. The size of points indicates the number of genes included in each gene set. **B** Volcano plot image of characterized gene expression from enriched categories in patients with Dmax ≥ 6 cm, as compared to patients with Dmax < 6 cm in CAR-T screening phase. The background color in the box represents which categories the gene belongs to. The black dashed line corresponds to *p* = 0.05. **C** Normalized expression of M2 macrophage, CAF, MDSC, and intermediate Tex of patients with Dmax ≥ 6 cm and Dmax < 6 cm in the CAR-T screening phase. BP, biological process; TME, tumor microenvironment; CAF, cancer-associated fibroblasts; MDSC, myeloid-derived suppressor cell; Tex, exhausted T cell

Gene Set Enrichment Analysis (GSEA) of RNA-sequencing data showed that immunosuppressive-related biological processes were enriched in patients with Dmax ≥ 6cm (Fig. 2). Tumor microenvironment (TME) analysis revealed higher levels of M2 macrophages, cancer-associated fibroblasts (CAF), myeloid-derived suppressor cell (MDSC), and intermediate exhausted T cells in these patients, suggesting an immunosuppressive microenvironment and a possible reason for CAR-T cell therapy failure in patients with high tumor burden. Accompanied by the increasing immunosuppressive cells within TME, the lipid metabolic, iron/copper ion transport, macrophage, granulocyte, monocyte chemotaxis, and autophagy pathways were significantly activated (Dmax ≥ 6cm). Under high tumor burden, tumor cells tend to recruit and activate more immunosuppressive cells, through metabolism alterations and subsequent induction of T-cell exhaustions [9, 10].

In summary, imaging metrics of 18F-FDG PET/CT, especially Dmax at the screening stage had the predictive value of clinical efficacy, progression, and death of CAR-T cell therapy, while the distance of the farthest lesions was associated with the occurrence of NT. Furthermore, we developed a prediction model combining M1 SUVmax and tTLG clearance rate to predict early progression for patients evaluated as PR/SD at M1 after CAR-T cell therapy. Immunosuppressive TME may serve as a possible mechanism for those patients who respond poorly to CAR-T cell therapy with high tumor burden.

## Supplementary Information

Supplementary Material 1.

Supplementary Material 2: Figure S1. ROC curves of screening-phase 18F-FDG PET/CT metrics with the 3-month response, PFS, and OS. (A-C) ROC curves of screening-phase 18F-FDG PET/CT metrics (Dmax, tMTV, tTLG, SUVmax, and sDmax) with PFS (A); OS (B), and 3-month response (C). (D) ROC curves of M1 18F-FDG PET/CT metrics after CAR-T cell therapy (M1 tMTV, M1 tTLG, and M1 SUVmax), (E–F) Δvalue (ΔtMTV, ΔtTLG, and ΔSUVmax) (E) and Δvalue / value^pre^ (ΔtMTV/tMTV^pre^, ΔtTLG/tTLG^pre^ and ΔSUVmax/SUVmax^pre^) (F) with PFS. ROC, receiver operating characteristic; SUVmax, maximum standardized uptake value; sDmax, the distance separating the two farthest lesions, standardized according to the body surface area.

Supplementary Material 3: Figure S2. The value of 18F-FDG PET/CT metrics in predicting early progression in PR/SD patients on M1 after CAR-T cell therapy. (A-B) PFS of patients evaluated as PR/SD on M1 after CAR-T cell therapy stratified with M1 SUVmax and ΔtTLG/tTLG^pre^ from RJ cohort (A) and Lyon cohort (B). (C-D) OS of patients evaluated as PR/SD on M1 after CAR-T cell therapy stratified with M1 SUVmax and ΔtTLG/tTLG^pre^ from RJ cohort (C) and Lyon cohort (D). (E) A prediction model combining M1 SUVmax and tTLG clearance rate to predict early progression for patients evaluated as PR/SD at M1 after CAR-T cell therapy. PR, partial response; SD, stable disease.

Supplementary Material 4:  Figure S3. Correlation of screening-phase 18F-FDG PET/CT metrics with CAR-T toxicity. (A-B) ROC curves of screening-phase 18F-FDG PET/CT metrics (Dmax, tMTV, tTLG, SUVmax, and sDmax) withCRS grade < 2 or CRS grade ≥ 2 (A) and no NT grade or any NT grade (B) in patients received axi-cel treatment. ROC curves of screening-phase 18F-FDG PET/CT metrics (Dmax, tMTV, tTLG, SUVmax and sDmax) with CRS grade < 2 or CRS grade ≥ 2 (C) and no NT grade or any NT grade (D) in patients received relma-cel treatment. (E) Occurrence of NT in patients stratified with screening-phase sDmax. (F) Correlation of screening-phase 18F-FDG PET/CT metrics with duration of CRS and NT. (G) Correlation of screening-phase 18F-FDG PET/CT metrics with onset day of CRS and NT. CRS, cytokine release syndrome; NT, neurotoxicity; Axi-cel, axicabtagene ciloleucel; relma-cel, relmacabtagene autoleucel.

Supplementary Material 5: Figure S4. Correlation of screening-phase 18F-FDG PET/CT metrics with CAR-T cell expansion. Correlation of screening-phase 18F-FDG PET/CT metrics with the duration of CAR-T Cmax (A), Tmax (B), and AUC_0-28d_ (C). Cmax, the peak CAR-T cell expansion value; Tmax, the days to peak expansion; AUC_0-28d_, expansion area under curve of day 0–28 after CAR-T cell therapy.

## Data Availability

The data used in this study are available from the corresponding author on reasonable request.

## Abbreviations

CAR-T: Chimeric antigen receptor T
LBCL: Large B-cell lymphoma
18F-FDG PET/CT: 18F-fluorodeoxyglucose positron emission tomography/computed tomography
tMTV: Total metabolic tumor volume
tTLG: Total lesion glycolysis
SUVmax: Maximum standardized uptake value
ORR: Objective remission rate
CR: Complete response
OS: Overall survival
PFS: Progression-free survival
CRS: Cytokine release syndrome
LDH: Lactate dehydrogenase
PR: Partial response
SD: Stable disease
NT: Neurotoxicity
Axi-cel: Axicabtagene ciloleucel
AUC: Area under curve
Relma-cel: Relmacabtagene autoleucel
GSEA: Gene Set Enrichment Analysis
TME: Tumor microenvironment
CAF: Cancer-associated fibroblasts
MDSC: Myeloid-derived suppressor cell

## References

[1] Neelapu SS, Jacobson CA, Ghobadi A, Miklos DB, Lekakis LJ, Oluwole OO, et al. Five-year follow-up of ZUMA-1 supports the curative potential of axicabtagene ciloleucel in refractory large B-cell lymphoma. Blood. 2023;141(19):2307–15. 36821768 10.1182/blood.2022018893 PMC10646788

[2] Leithner D, Flynn JR, Devlin SM, Mauguen A, Fei T, Zeng S, et al. Conventional and novel [(18)F]FDG PET/CT features as predictors of CAR-T cell therapy outcome in large B-cell lymphoma. J Hematol Oncol. 2024;17(1):21. 38649972 10.1186/s13045-024-01540-x PMC11035117

[3] Gui J, Li M, Xu J, Zhang X, Mei H, Lan X. [18F]FDG PET/CT for prognosis and toxicity prediction of diffuse large B-cell lymphoma patients with chimeric antigen receptor T-cell therapy. Eur J Nucl Med Mol Imaging. 2024;51:2308–19. 10.1007/s00259-024-06667-0 38467921

[4] Sesques P, Tordo J, Ferrant E, Safar V, Wallet F, Dhomps A, et al. Prognostic Impact of 18F-FDG PET/CT in Patients With Aggressive B-Cell Lymphoma Treated With Anti-CD19 Chimeric Antigen Receptor T Cells. Clin Nucl Med. 2021;46(8):627–34. 34115706 10.1097/RLU.0000000000003756

[5] Breen WG, Hathcock MA, Young JR, Kowalchuk RO, Bansal R, Khurana A, et al. Metabolic characteristics and prognostic differentiation of aggressive lymphoma using one-month post-CAR-T FDG PET/CT. J Hematol Oncol. 2022;15(1):36. 35346315 10.1186/s13045-022-01256-w PMC8962609

[6] Breen WG, Young JR, Hathcock MA, Kowalchuk RO, Thorpe MP, Bansal R, et al. Metabolic PET/CT analysis of aggressive Non-Hodgkin lymphoma prior to Axicabtagene Ciloleucel CAR-T infusion: predictors of progressive disease, survival, and toxicity. Blood Cancer J. 2023;13(1):127. 37591834 10.1038/s41408-023-00895-7 PMC10435575

[7] Ababneh HS, Ng AK, Abramson JS, Soumerai JD, Takvorian RW, Frigault MJ, et al. Metabolic parameters predict survival and toxicity in chimeric antigen receptor T-cell therapy-treated relapsed/refractory large B-cell lymphoma. Hematol Oncol. 2024;42(1):e3231. 37795759 10.1002/hon.3231

[8] Wang J, Hu Y, Yang S, Wei G, Zhao X, Wu W, et al. Role of Fluorodeoxyglucose Positron Emission Tomography/Computed Tomography in Predicting the Adverse Effects of Chimeric Antigen Receptor T Cell Therapy in Patients with Non-Hodgkin Lymphoma. Biol Blood Marrow Transplant. 2019;25(6):1092–8. 30769193 10.1016/j.bbmt.2019.02.008

[9] Cerchietti L. Genetic mechanisms underlying tumor microenvironment composition and function in diffuse large B-cell lymphoma. Blood. 2024;143(12):1101–11. 38211334 10.1182/blood.2023021002 PMC10972714

[10] Yan Z, Li L, Fu D, Wu W, Qiao N, Huang Y, et al. Immunosuppressive tumor microenvironment contributes to tumor progression in diffuse large B-cell lymphoma upon anti-CD19 chimeric antigen receptor T therapy. Front Med. 2023;17(4):699–713. 37060525 10.1007/s11684-022-0972-8

