# Supplementary material for: 18F-FDG PET/CT metrics-based stratification of large B-cell lymphoma receiving CAR-T cell therapy: immunosuppressive tumor microenvironment as a negative prognostic indicator in patients with high tumor burden
Source: Biomark Res. 2024 Sep 14;12:104. doi: 10.1186/s40364-024-00650-5 (PMC11401356; doi:10.1186/s40364-024-00650-5)
Supplement: Supplementary file 1 — Supplementary Material 1. [file 40364_2024_650_MOESM1_ESM.docx]

**Material and methods**

**Study population**

Patients from Shanghai Ruijin Hospital (hereafter referred to as RJ cohort) who met the following criteria were included: received chimeric antigen receptor T (CAR-T) cell therapy as part of B-cell lymphoma treatment between January 1, 2018, and March 31, 2023, and undergone 18F-fluorodeoxyglucose positron emission tomog­raphy/computed tomography (18F-FDG PET/CT) scan at screening stage, 1- and 3-months after CAR-T cell infusion. Infusion and post-infusion management were based on institutional protocols.

Histological diagnosis was established based on revised 2016 World Health Organization (WHO) classification (4th edition)(1). A variety of clinical data including gender, age, number of extranodal involvement, serum lactic dehydrogenase (LDH), performance status (Eastern Cooperative Oncology Group, ECOG), Ann Arbor stage, international prognostic index (IPI) score, response to first-line therapy, progression-free survival (PFS) and overall survival (OS) were collected, with the last follow-up date of October 1, 2023. As for pathological subtypes, germinal center B-cell like (GCB) or non-GCB subgroups were determined using the Hans classification(2), with 30% cut-off values for CD10, BCL-6, and MUM-1. For BCL-2/MYC double expressor lymphoma (DEL), cut-off values for BCL-2 and MYC were 50% and 40%, respectively(3, 4). The study was approved by Shanghai Ruijin Hospital Ethics Board and informed consent was obtained from all patients in accordance with the Declaration of Helsinki.

Patients from Lyon Sud Hospital (hereafter referred to as Lyon cohort) were described in a published manuscript(5). This cohort was used to validate a prediction model for early prediction of prognosis in partial response (PR)/stable disease (SD) patients at M1 after CAR-T cell therapy.

**18F-FDG PET/CT analysis**

All patients received 18F-FDG PET/CT scan at screening stage, 1 month and 3 months after CAR-T cell infusion (Figure 1A). All 18F-FDG PET/CT images were reviewed and analyzed on a Syngo.via workstation (VB20A, Siemens Healthcare, Erlangen, Germany). Semi-quantitative analysis of tumor metabolic activity was obtained using standard uptake value normalized to body weight. All parameters were assessed in 3-dimensional volumes. A volume of interest (VOI) isocontour of the tumor region were applied, and semi-quantitative PET imaging analysis were carried out after the lesion delineation procedures. The above-mentioned procedures were carried out by two experienced nuclear medicine physicians separately to double confirm the correct inclusion and reproducible metrics measurements of the lymphoma. A designated operator delineated and then segmented the region according to the threshold of 41% of the standardized uptake value (SUVmax) of each lesion, in accordance with the recommendations of the European Association of Nuclear Medicine(6). Each VOI generated a SUVmax, a metabolic tumor volume (MTV), and a total lesion glycolysis (TLG) value. The values of the following features were obtained for each PET scan: maximum diameter of the largest lesion (Dmax), distance separating the two farthest lesions, standardized by body surface area, total MTV (tMTV), total TLG (tTLG) and SUVmax. Changes of each metrics from screening stage to 1 months were also calculated, and Δvalue = [value at screening stage] - [value at M1], Δvalue / value^pre^ = Δvalue / (value at screening stage).

**Assessment of response and toxicity**

The response to CAR-T treatment was defined by the 2014 Lugano criteria through the reading of follow-up 18F FDG-PET/CT scans performed 1 month and 3 months after CAR-T cell infusion(7-9). PFS was defined as the time between the date of CAR-T cell infusion and disease relapse/progression or death from any cause. OS was defined as the time between the date of injection of CAR-T cells and death from any cause. The occurrences of cytokine release syndrome (CRS) and neurotoxicity (NT) were collected in the medical records of patients. Severity was scored from 1 to 4 based on the classification of the American Society for Transplantation and Cellular Therapy(10). The occurrences of CRS and NT were collected in the medical records of patients.

**Detection of CAR-T cell expansion**

CD19 CAR-expressing T cells were determined using a combination of the following three antibodies included in the CD19 CAR detection reagent: PE-labeled rabbit anti-mouse FMC63 scFv monoclonal antibody (BioSwan Laboratories, Shanghai, China), APC-labeled CD3 (Becton Dickinson, San Jose, CA, USA), and QB500-labeled CD45 (QuantoBio, Tianjin, China). The samples were analyzed using a FACSLyric cytometer (Becton Dickinson) according to the manufacturer’s instructions. At least 10000 lymphocytes with low SSC and high CD45 expression were acquired, and data were analyzed with the KALUZA software (Beckman Coulter, Brea, CA, USA). The CD19 CAR-expressing T cells were defined as those that were double-positive for FMC63 and CD3 in a gated population of lymphocytes and were quantified as the percentage of the total number of white blood cells. The absolute count of CD19 CAR-expressing T cells was calculated by multiplying the white blood cell count with the percentage of the calculated double-positive cells.

**RNA-sequencing**

Total RNA was extracted from frozen tumor tissue samples by Trizol and RNeasy Mini Kit (QIAGEN), and the integrity of total RNA was evaluated by RNA 6000 Nano Kit on Aligent 2100 Bioanalyzer. Read pairs were aligned to Refseq hg19 with Burrows-Wheeler Aligner version 0.7.13-r1126. Transcript counts table files were generated via HTSeq(11). Potential false positive results were excluded via visual inspection. Bioinformatic analyses were performed through R 3.5.1, with R package “sva” for batch effect removal. Raw reads were normalized, and differentially expressed genes were obtained with R package “limma” (v3.38.3).

**Gene Set Enrichment Analysis (GSEA)**

GSEA was conducted with GSEA v4.1.0 software and Molecular Signature Database (MSigDB) v7.4(12). The metric for ranking genes was Signal2Noise by default. Phenotypes contained at least 7 samples were labeled permutation type. Based on GSEA team recommendation ([*http://www.broadinstitute.org/gsea*](http://www.broadinstitute.org/gsea)), statistical significance of enrichment score was assessed with permutation being set up at 1,000. Enriched pathways were considered statistically significant with P value under 0.05.

**Tumor microenvironment analysis**

Gene expression signatures of variable cells within the tumor microenvironment (TME) was based on the average expression of selected genes: M2 macrophage (C1QA, C1QB, C1QC, SLC40A1, APOC1, APOE, MMP9, GPNMB, CHI3L1), CAF (COL1A1, COL3A1), MDSC (CCR2, CXCR2, C5ar1, IL1B, CSF3R, IFITM1, ARG2, WFDC17, CD84) and intermediate Tex (EOMES, CCR5, GZMA, GZMK, HLA-DRB1, IFNG) (13, 14).

**Statistical analyses**

Baseline patient characteristics and PET/CT metrics were summarized as counts and percentages, median and interquartile range, or mean and standard deviation. The optimal thresholds of PET/CT metabolic metrics for prognosis were quantitatively determined using the area under the receiver operating characteristic (ROC) curve. When appropriate, continuous PET values were dichotomized to create scientifically appropriate groups (e.g., Dmax<6cm vs. ≥6cm). Survival analyses were estimated using the Kaplan-Meier method and compared by log-rank test. Hazard ratios (HRs) and 95% confidence intervals (CIs) were reported. Rank correlation analysis was used to determine correlation coefficient (R^2^). The predictive value of features on PFS were analyzed using univariate and stepwise multivariate logistic regression. Associations between the toxicity or CAR-T expansion and imaging metrics in rank correlation analysis were analyzed using a multinomial logistic regression model. Statistical significance was defined as *P*<0.05. GraphPad Prism v7 (USA) was used for graphing and visualizing the data. The above statistical analyses were performed by Statistical Package for the Social Sciences (SPSS) 26.0 software (SPSS Inc., Chicago, IL).

**References**

1. Swerdlow SH, editor WHO Classification of Tumours of Haematopoietic and Lymphoid Tissues2017.

2. Hans CP, Weisenburger DD, Greiner TC, Gascoyne RD, Delabie J, Ott G, et al. Confirmation of the molecular classification of diffuse large B-cell lymphoma by immunohistochemistry using a tissue microarray. Blood. 2004;103(1):275-82.

3. Horn H, Ziepert M, Becher C, Barth TF, Bernd HW, Feller AC, et al. MYC status in concert with BCL2 and BCL6 expression predicts outcome in diffuse large B-cell lymphoma. Blood. 2013;121(12):2253-63.

4. Staiger AM, Ziepert M, Horn H, Scott DW, Barth TFE, Bernd HW, et al. Clinical Impact of the Cell-of-Origin Classification and the MYC/ BCL2 Dual Expresser Status in Diffuse Large B-Cell Lymphoma Treated Within Prospective Clinical Trials of the German High-Grade Non-Hodgkin's Lymphoma Study Group. J Clin Oncol. 2017;35(22):2515-26.

5. Sesques P, Tordo J, Ferrant E, Safar V, Wallet F, Dhomps A, et al. Prognostic Impact of 18F-FDG PET/CT in Patients With Aggressive B-Cell Lymphoma Treated With Anti-CD19 Chimeric Antigen Receptor T Cells. Clin Nucl Med. 2021;46(8):627-34.

6. Meignan M, Sasanelli M, Casasnovas RO, Luminari S, Fioroni F, Coriani C, et al. Metabolic tumour volumes measured at staging in lymphoma: methodological evaluation on phantom experiments and patients. Eur J Nucl Med Mol Imaging. 2014;41(6):1113-22.

7. Cheson BD, Fisher RI, Barrington SF, Cavalli F, Schwartz LH, Zucca E, et al. Recommendations for initial evaluation, staging, and response assessment of Hodgkin and non-Hodgkin lymphoma: the Lugano classification. J Clin Oncol. 2014;32(27):3059-68.

8. Barrington SF, Mikhaeel NG, Kostakoglu L, Meignan M, Hutchings M, Mueller SP, et al. Role of imaging in the staging and response assessment of lymphoma: consensus of the International Conference on Malignant Lymphomas Imaging Working Group. J Clin Oncol. 2014;32(27):3048-58.

9. O JH, Lodge MA, Wahl RL. Practical PERCIST: A Simplified Guide to PET Response Criteria in Solid Tumors 1.0. Radiology. 2016;280(2):576-84.

10. Lee DW, Santomasso BD, Locke FL, Ghobadi A, Turtle CJ, Brudno JN, et al. ASTCT Consensus Grading for Cytokine Release Syndrome and Neurologic Toxicity Associated with Immune Effector Cells. Biol Blood Marrow Transplant. 2019;25(4):625-38.

11. Anders S, Pyl PT, Huber W. HTSeq--a Python framework to work with high-throughput sequencing data. Bioinformatics. 2015;31(2):166-9.

12. Subramanian A, Tamayo P, Mootha VK, Mukherjee S, Ebert BL, Gillette MA, et al. Gene set enrichment analysis: a knowledge-based approach for interpreting genome-wide expression profiles. Proc Natl Acad Sci U S A. 2005;102(43):15545-50.

13. Zheng L, Qin S, Si W, Wang A, Xing B, Gao R, et al. Pan-cancer single-cell landscape of tumor-infiltrating T cells. Science. 2021;374(6574):abe6474.

14. Ye X, Wang L, Nie M, Wang Y, Dong S, Ren W, et al. A single-cell atlas of diffuse large B cell lymphoma. Cell Rep. 2022;39(3):110713.
