# Supplementary figures and images for: 18F-FDG PET/CT metrics-based stratification of large B-cell lymphoma receiving CAR-T cell therapy: immunosuppressive tumor microenvironment as a negative prognostic indicator in patients with high tumor burden
Source: Biomark Res. 2024 Sep 14;12:104. doi: 10.1186/s40364-024-00650-5 (PMC11401356; doi:10.1186/s40364-024-00650-5)

**Supplementary figure 1**

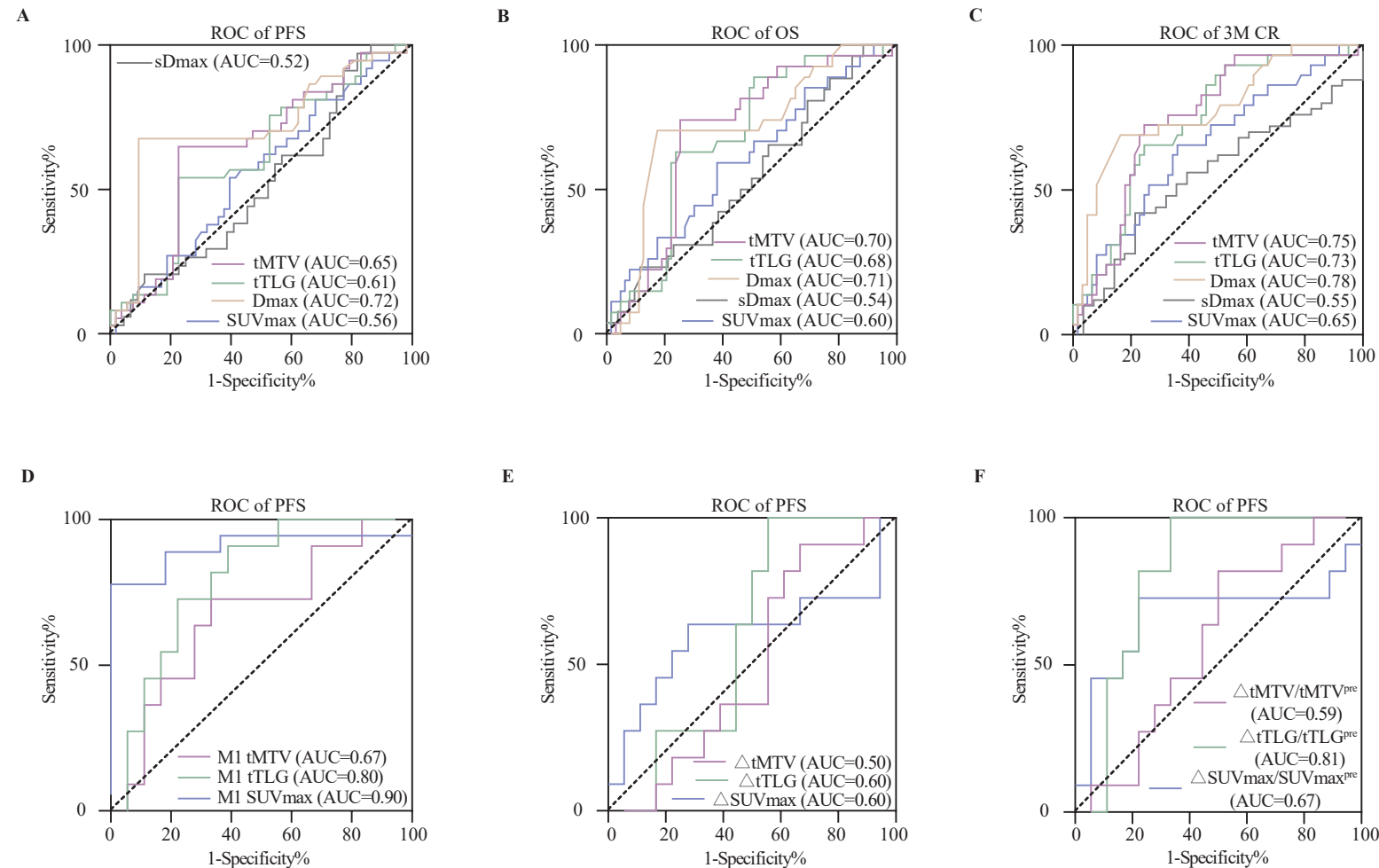

Supplement: Supplementary file 2 — Supplementary Material 2: Figure S1. ROC curves of screening-phase 18F-FDG PET/CT metrics with the 3-month response, PFS, and OS. (A-C) ROC curves of screening-phase 18F-FDG PET/CT metrics (Dmax, tMTV, tTLG, SUVmax, and sDmax) with PFS (A); OS (B), and 3-month response (C). (D) ROC curves of M1 18F-FDG PET/CT metrics after CAR-T cell therapy (M1 tMTV, M1 tTLG, and M1 SUVmax), (E–F) Δvalue (ΔtMTV, ΔtTLG, and ΔSUVmax) (E) and Δvalue / valuepre (ΔtMTV/tMTVpre, ΔtTLG/tTLGpre and ΔSUVmax/SUVmaxpre) (F) with PFS. ROC, receiver operating characteristic; SUVmax, maximum standardized uptake value; sDmax, the distance separating the two farthest lesions, standardized according to the body surface area. [file 40364_2024_650_MOESM2_ESM.pdf]

Supplementary figure 2

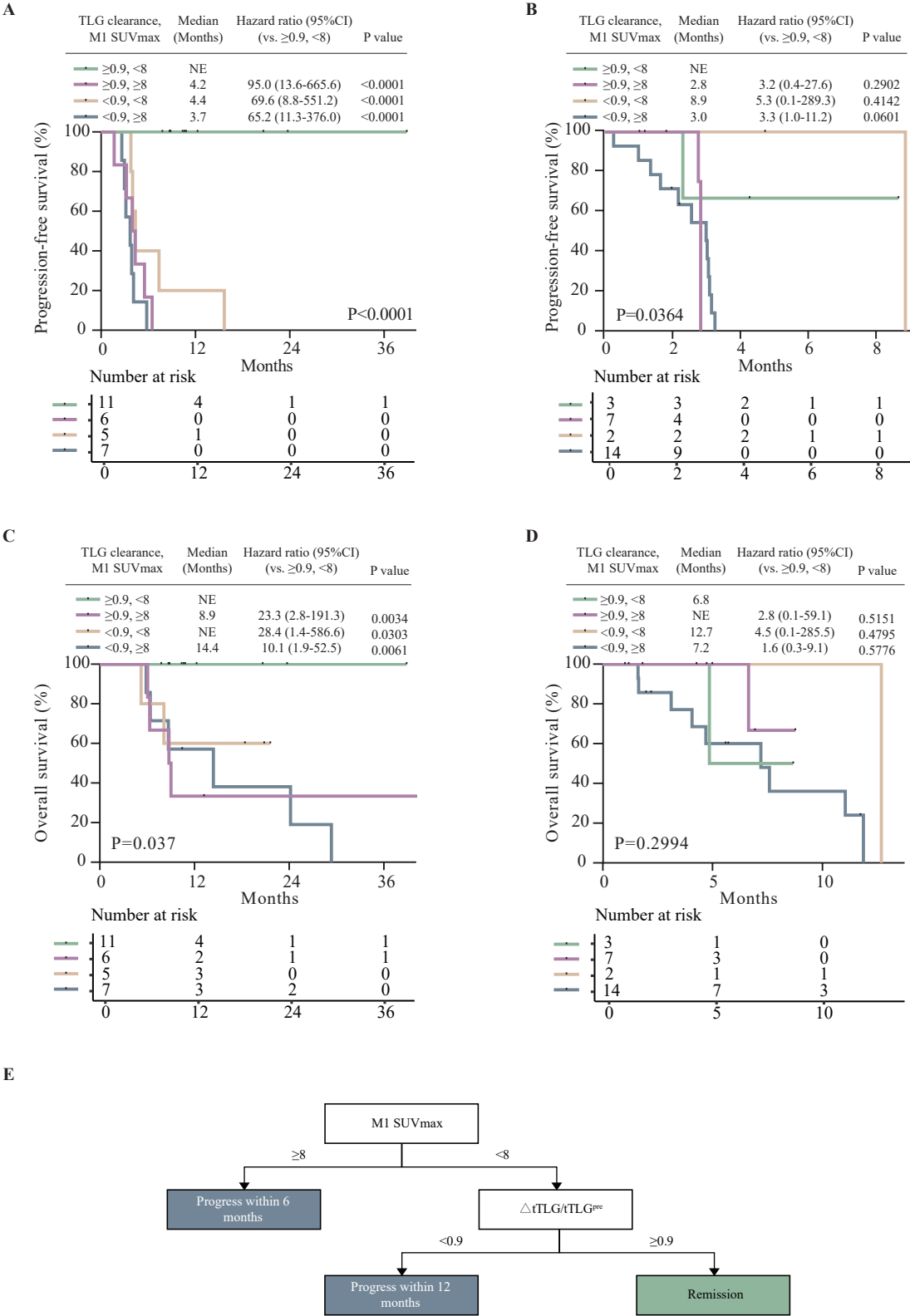

Supplement: Supplementary file 3 — Supplementary Material 3: Figure S2. The value of 18F-FDG PET/CT metrics in predicting early progression in PR/SD patients on M1 after CAR-T cell therapy. (A-B) PFS of patients evaluated as PR/SD on M1 after CAR-T cell therapy stratified with M1 SUVmax and ΔtTLG/tTLGpre from RJ cohort (A) and Lyon cohort (B). (C-D) OS of patients evaluated as PR/SD on M1 after CAR-T cell therapy stratified with M1 SUVmax and ΔtTLG/tTLGpre from RJ cohort (C) and Lyon cohort (D). (E) A prediction model combining M1 SUVmax and tTLG clearance rate to predict early progression for patients evaluated as PR/SD at M1 after CAR-T cell therapy. PR, partial response; SD, stable disease. [file 40364_2024_650_MOESM3_ESM.pdf]

**Supplementary figure 3**

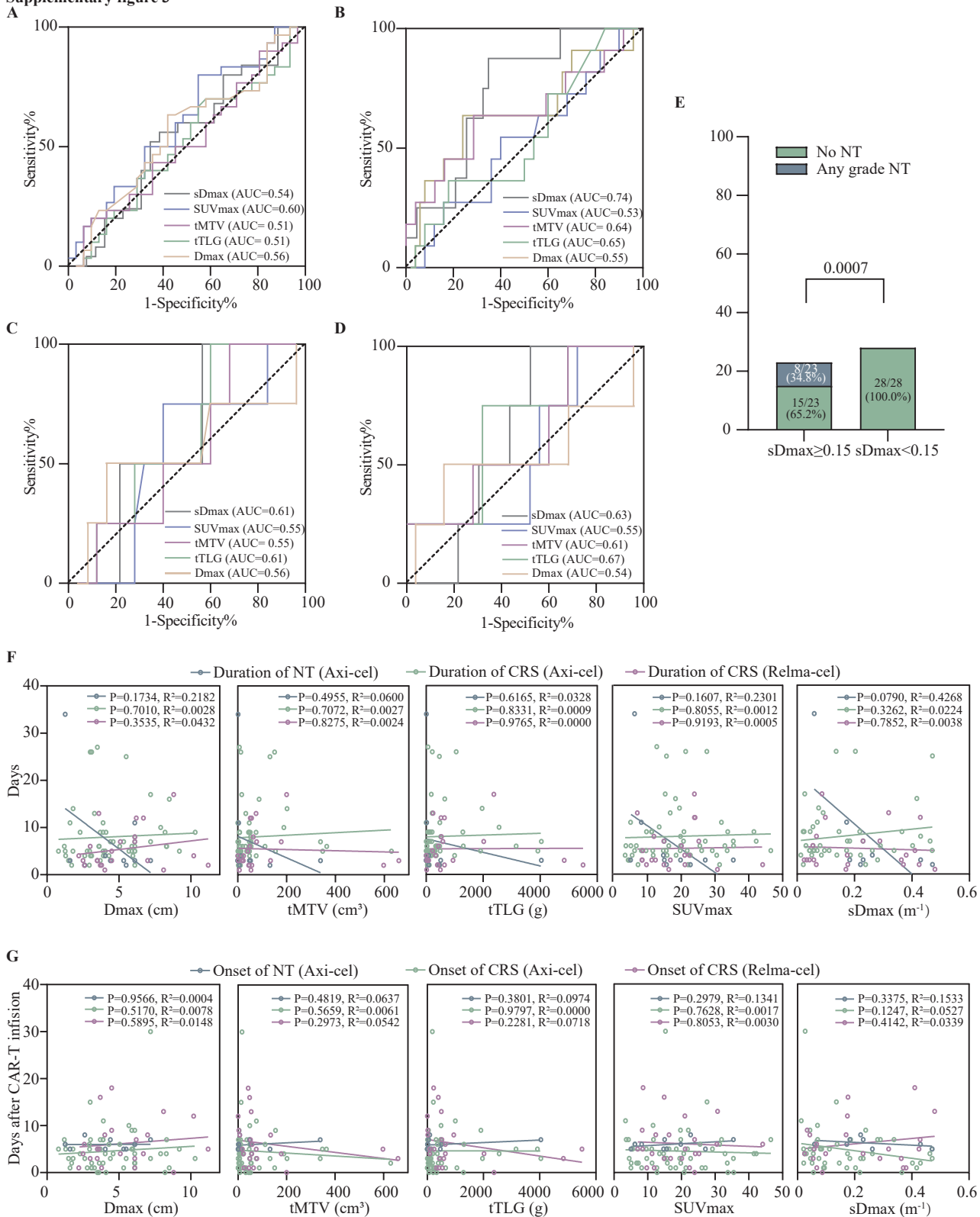

Supplement: Supplementary file 4 — Supplementary Material 4: Figure S3. Correlation of screening-phase 18F-FDG PET/CT metrics with CAR-T toxicity. (A-B) ROC curves of screening-phase 18F-FDG PET/CT metrics (Dmax, tMTV, tTLG, SUVmax, and sDmax) withCRS grade < 2 or CRS grade ≥ 2 (A) and no NT grade or any NT grade (B) in patients received axi-cel treatment. ROC curves of screening-phase 18F-FDG PET/CT metrics (Dmax, tMTV, tTLG, SUVmax and sDmax) with CRS grade < 2 or CRS grade ≥ 2 (C) and no NT grade or any NT grade (D) in patients received relma-cel treatment. (E) Occurrence of NT in patients stratified with screening-phase sDmax. (F) Correlation of screening-phase 18F-FDG PET/CT metrics with duration of CRS and NT. (G) Correlation of screening-phase 18F-FDG PET/CT metrics with onset day of CRS and NT. CRS, cytokine release syndrome; NT, neurotoxicity; Axi-cel, axicabtagene ciloleucel; relma-cel, relmacabtagene autoleucel. [file 40364_2024_650_MOESM4_ESM.pdf]

Supplementary figure 4

A

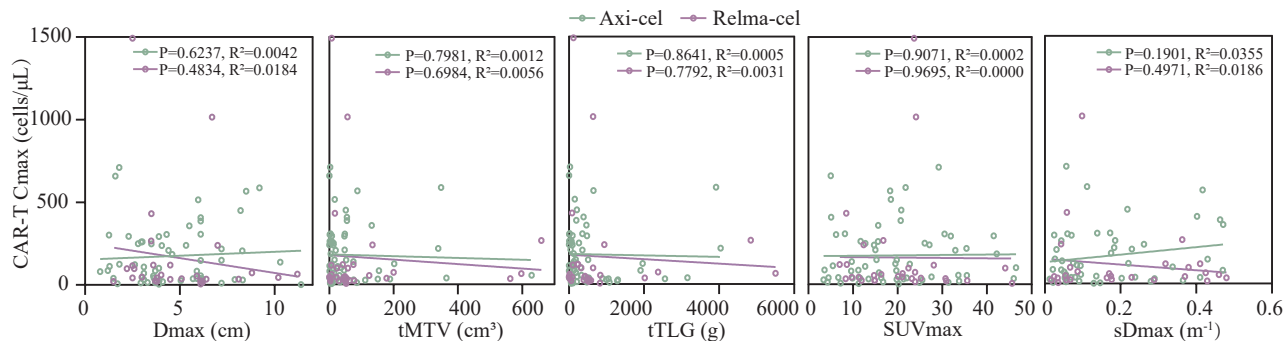

B

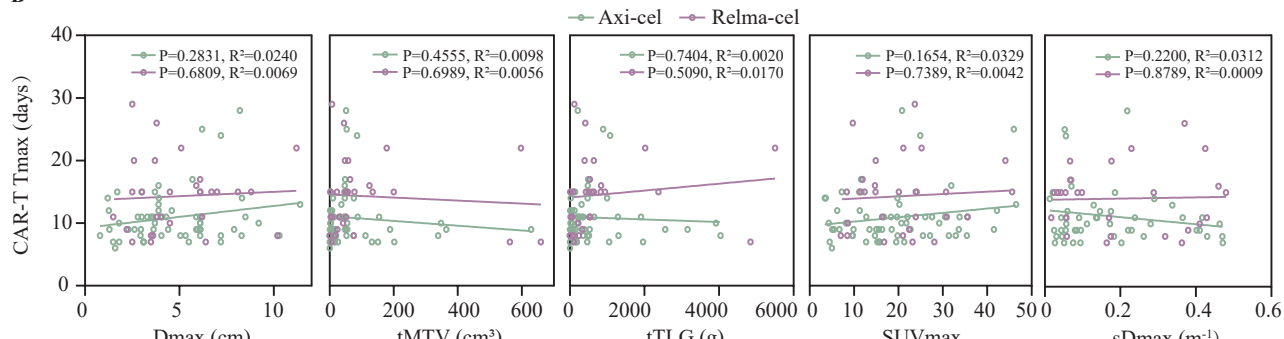

C

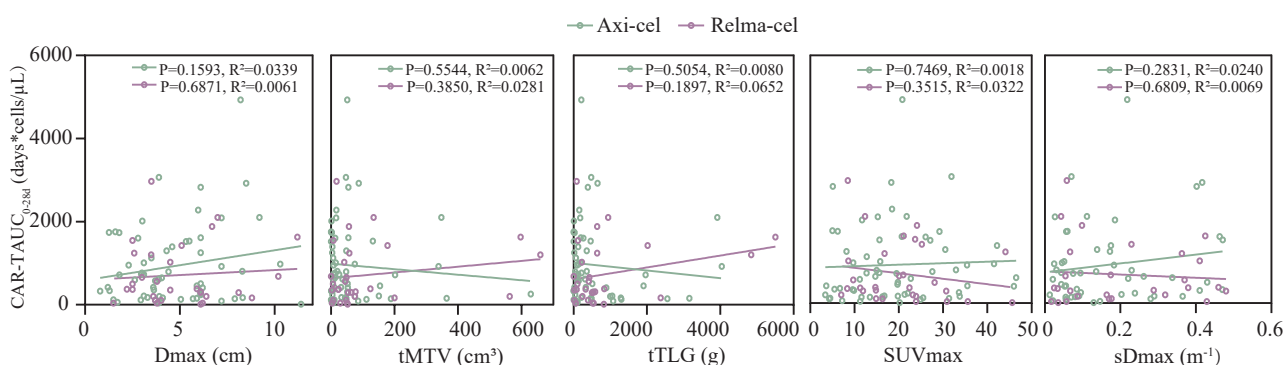

Supplement: Supplementary file 5 — Supplementary Material 5: Figure S4. Correlation of screening-phase 18F-FDG PET/CT metrics with CAR-T cell expansion. Correlation of screening-phase 18F-FDG PET/CT metrics with the duration of CAR-T Cmax (A), Tmax (B), and AUC0-28d (C). Cmax, the peak CAR-T cell expansion value; Tmax, the days to peak expansion; AUC0-28d, expansion area under curve of day 0–28 after CAR-T cell therapy. [file 40364_2024_650_MOESM5_ESM.pdf]
